# A regulatory network underlying idiopathic pulmonary fibrosis

**DOI:** 10.3389/fimmu.2026.1919175

**Published:** 2026-08-20

**Authors:** Ganggang Li, Xiaochuan Pan, Yuanyuan Wang, Peishu Lan, Jie Li, Xuanyu Wu, Yanyun He, Hongdan Wen, Zhitian Cheng, Quanyu Du, Wenbin Wu

**Affiliations:** 1 Hospital of Chengdu University of Traditional Chinese Medicine, School of Clinical Medicine, Chengdu University of Traditional Chinese Medicine, Chengdu, China; 2 Affiliated Tumor Hospital of Xinjiang Medical University, Urumqi, China

**Keywords:** idiopathic pulmonary fibrosis, mendelian randomization, multi-omics integration, regulatory network, single-cell and spatial transcriptomics

## Abstract

**Background:**

Idiopathic pulmonary fibrosis (IPF) is a progressive interstitial lung disease in which genetic susceptibility interacts with epithelial, immune, and mesenchymal remodeling. Although the chromosome 11p15.5 locus contains established IPF susceptibility signals near MUC5B and TOLLIP, the broader regulatory architecture of this region remains incompletely resolved.

**Methods:**

We integrated IPF genome-wide association study summary statistics with methylation, expression, and protein quantitative trait loci using summary-data-based Mendelian randomization (SMR). SMR-prioritized candidates were evaluated in independent transcriptomic and methylation cohorts and further contextualized using microRNA, transcription-factor, protein-interaction, machine-learning, single-cell, and spatial transcriptomic analyses. Fibrosis-associated expression patterns were assessed in a bleomycin-induced pulmonary fibrosis rat model.

**Results:**

The analyses recovered the established MUC5B and TOLLIP signals and prioritized BRSK2 as a comparatively underexplored candidate supported by eQTL-based SMR and independent molecular evidence. The BRSK2 pQTL association did not pass the HEIDI test and was therefore not interpreted as convergent protein-level genetic evidence. Network analyses linked BRSK2 to cell-cycle, metabolic-stress, and senescence-related programs, while cross-cohort machine learning prioritized FOXA2, CDC25B, and NFE2 as informative network features. Single-cell and spatial analyses localized BRSK2 preferentially to fibroblast and myofibroblast compartments and to regions with greater histological fibrosis severity. In fibrotic rat lungs, BRSK2 expression increased, whereas FOXA2 and CDC25B decreased at the transcript and protein levels.

**Conclusions:**

These findings refine the molecular landscape of the chromosome 11p15.5 IPF susceptibility locus and prioritize BRSK2 as a candidate component of an IPF-associated profibrotic fibroblast state. Its causal contribution, direct regulatory relationships, and therapeutic tractability require targeted mechanistic validation.

## 1 Introduction

Idiopathic pulmonary fibrosis (IPF) is a progressive and ultimately fatal interstitial lung disease characterized by persistent fibroblast activation, excessive extracellular matrix deposition, and irreversible disruption of the alveolar architecture (1). Current models of IPF pathogenesis emphasize maladaptive interactions among injured epithelial cells, immune populations, fibroblasts, and myofibroblasts within a spatially heterogeneous lung microenvironment (2). However, the molecular mechanisms through which inherited susceptibility is translated into cell-type-specific transcriptional programs and persistent mesenchymal remodeling remain incompletely understood.

Genetic studies have substantially advanced the understanding of IPF susceptibility and have repeatedly implicated the chromosome 11p15.5 region, including variants near mucin 5B (MUC5B) and Toll interacting protein (TOLLIP) (3, 4). Nevertheless, assigning disease-associated variants to their functional effector genes remains challenging because most risk variants reside in non-coding regions, may act through tissue- and cell-state-specific regulatory mechanisms, and do not necessarily regulate the nearest gene. The broader regulatory architecture of the 11p15.5 locus, particularly beyond the established MUC5B and TOLLIP signals, therefore remains insufficiently resolved. Integration of genome-wide association study data with molecular quantitative trait loci, including methylation, expression, and protein QTLs, provides a means of prioritizing genes whose molecular traits share genetic associations with disease risk (5). Summary-data-based Mendelian randomization (SMR) is useful in this context, although its interpretation remains sensitive to linkage disequilibrium, heterogeneity, tissue context, and instrument validity and therefore requires corroboration across complementary molecular and experimental evidence (6).

In the present study, we applied a multi-layer integrative framework to systematically interrogate the regulatory landscape of the chromosome 11p15.5 IPF susceptibility locus. Genetic prioritization across multiple QTL layers was combined with bulk transcriptomic profiling, regulatory-network and machine-learning analyses, single-cell and spatial transcriptomics, and experimental validation. We aimed to distinguish established susceptibility signals from additional candidate regulators, define their cellular and spatial contexts within fibrotic lungs, and assess the consistency of the resulting associations across molecular layers. This framework recovered the established MUC5B and TOLLIP signals and prioritized BRSK2 as a candidate associated with fibroblast and myofibroblast remodeling, while recognizing that its direct functional and causal role requires further investigation.

## 2 Methods

### 2.1 Data sources and study design

All datasets analyzed in this study were obtained from publicly available databases, including the GWAS Catalog, Gene Expression Omnibus (GEO), and ArrayExpress. The integrated framework incorporated IPF GWAS summary statistics, molecular quantitative trait locus datasets, bulk transcriptomic and methylation datasets, microRNA expression profiles, single-cell RNA-sequencing data, and spatial transcriptomic data (7–18). The accession numbers, profiling platforms, sample composition, and corresponding references are provided in **Supplementary Table 1**.

### 2.2 Summary-data-based Mendelian randomization analysis

Summary-data-based Mendelian randomization (SMR) analysis was performed to evaluate associations between genetically regulated molecular traits and IPF susceptibility. IPF GWAS summary statistics were separately integrated with quantitative trait locus datasets derived from DNA methylation (mQTL), gene expression (eQTL), and protein abundance (pQTL). For each molecular trait, QTL variants meeting a significance threshold of *P_QTL_* < 1×10^-^^5^ were considered for instrumental-variable selection. SMR associations were evaluated independently within each molecular layer, and multiple testing was controlled using the Benjamini–Hochberg procedure. Associations with an FDR-adjusted *P_SMR_* < 0.05 were considered statistically significant. The heterogeneity in dependent instruments (HEIDI) test was applied to distinguish associations consistent with a shared genetic signal from those potentially attributable to linkage disequilibrium or allelic heterogeneity. Associations with *P_HEIDI_*> 0.01 were considered to pass the HEIDI test, whereas associations with *P*_HEIDI_≤ 0.01 were considered heterogeneous and were not interpreted as evidence of a shared causal variant (19). The SMR framework was additionally applied to examine shared genetic regulation between DNA methylation and gene expression through mQTL–eQTL cross-mapping. All analyses were conducted using SMR software version 1.3.1 (5).

### 2.3 Independent validation in transcriptomic and methylation cohorts

Candidate genes and CpG sites prioritized through the SMR analyses were evaluated in independent transcriptomic and methylation datasets. Microarray expression and methylation datasets were analyzed using the limma package, whereas high-throughput RNA-sequencing datasets were analyzed using DESeq2. Differential gene expression and DNA methylation between IPF and control samples were assessed separately within each dataset. Multiple comparisons were corrected using the Benjamini–Hochberg procedure, and features with an adjusted *P* < 0.05 were considered statistically significant. The direction and consistency of molecular alterations across independent datasets were considered when evaluating supporting evidence for the SMR-prioritized candidates.

### 2.4 Regulatory network construction and functional enrichment

To characterize the regulatory architecture surrounding the SMR-prioritized genes, an integrated network incorporating microRNAs, transcription factors (TFs), and protein–protein interactions (PPIs) was constructed. Candidate microRNAs targeting the core genes were predicted using miRDB and subsequently evaluated in independent IPF-related microRNA datasets. Candidate TFs were identified using the hTFtarget database and evaluated in bulk transcriptomic datasets. Associations between TFs and candidate target genes were assessed by Pearson correlation analysis using the GSE213001 dataset. TF–target pairs with *P* < 0.05 and |r| > 0.4 were retained. Protein-interaction partners were obtained from the STRING database version 12.0 and evaluated using independent transcriptomic datasets. Supported microRNA–target, TF–target, and protein-interaction relationships were integrated with the SMR-prioritized genes and visualized using Cytoscape version 3.10. KEGG pathway enrichment analysis was performed using the clusterProfiler package, and terms with an adjusted value *P* < 0.05 were considered significantly enriched.

### 2.5 Machine-learning prioritization within the BRSK2-associated network

Machine-learning analyses were performed to prioritize informative regulatory features within the integrated BRSK2-associated network using gene-expression profiles from GSE134692, GSE231693, and GSE213001. Candidate features were restricted to genes derived from the integrated multi-omics regulatory network before model fitting. Multiple regularized-regression, random-forest, boosting, support-vector, discriminant-analysis, and ensemble configurations were evaluated, and model discrimination was summarized by the area under the receiver operating characteristic curve (AUC) across the three cohorts. SHapley Additive exPlanations (SHAP) analysis was applied to the selected model to quantify the contribution of individual features. The primary aim of this framework was biological feature prioritization rather than development of a clinical diagnostic classifier.

### 2.6 Single-cell and spatial transcriptomic analyses

Single-cell RNA-sequencing datasets from healthy controls and patients with IPF were integrated using Seurat (v5.1.0) (20). Cells with fewer than 500 detected features or more than 10% mitochondrial transcripts were excluded, leaving 320, 616 cells for downstream analysis. Data were log-normalized, the 3, 000 most variable genes were used for principal component analysis, and Harmony (v1.2.3) was applied to reduce inter-study technical variation (21). Cells were clustered using a Louvain-based approach and annotated according to canonical markers and published lung cell atlases. Marker dot plots and selected UMAP feature plots were used to support cell-type annotation and characterize the distributions of BRSK2 and fibrosis-associated genes. UMAPs colored by study, donor, and disease condition, together with study-specific cell-type contributions, were used to assess potential cohort or donor segregation. Condition-stratified single-cell plots were descriptive, and individual cells were not treated as independent biological replicates.

Exploratory pseudotime analysis was performed using Monocle2 (v2.36.0) to descriptively visualize gene-expression patterns across computationally ordered cellular states (22). Because the integrated atlas contained heterogeneous lung cell populations, pseudotime was not interpreted as evidence of direct lineage relationships or chronological disease progression. Spatial transcriptomic data from E-MTAB-14121 were analyzed using the spatial functions implemented in Seurat. The regional categories were adopted from the original dataset annotations and were not independently reclassified in the present study. Control tissue (B0) was obtained from four healthy donors. For each of four patients with IPF, three spatially distinct peripheral lung tissue blocks were selected by histological inspection to represent mild (B1), moderate (B2), and severe (B3) regional fibrosis. A total of 25 Visium sections were analyzed, comprising six B0, seven B1, six B2, and six B3 sections, including replicate sections from selected tissue blocks. Thus, the independent donor numbers were four controls and four patients with IPF, whereas the reported B0–B3 sample numbers refer to tissue sections. The B1–B3 categories were interpreted as cross-sectional regional fibrosis-severity states within the same IPF lungs rather than longitudinal disease stages.

### 2.7 Virtual screening and molecular dynamics simulations

Candidate compounds potentially targeting BRSK2 and its network-associated proteins were collected from ChEMBL (23), the Therapeutic Target Database(TTD) (24), PharmSnap, and published literature. Pharmacokinetic properties were evaluated using SwissADME, and compounds with high predicted gastrointestinal absorption and a bioavailability score of at least 0.55 were retained. Putative compound targets were assessed using SwissTargetPrediction and integrated with the BRSK2-associated regulatory network to prioritize compounds with potential relevance to BRSK2, CDC25B, and WEE1.

Molecular docking was performed using AutoDock Vina to estimate the binding affinities and interaction modes of the prioritized compounds with BRSK2 and selected network-associated proteins. The highest-ranking protein–ligand complexes were subsequently subjected to 100-ns molecular dynamics simulations using GROMACS. Complex stability and conformational behavior were evaluated using the root-mean-square deviation (RMSD), root-mean-square fluctuation (RMSF), radius of gyration (Rg), and hydrogen-bond occupancy. Relative binding free energies were further estimated using the molecular mechanics/Poisson–Boltzmann surface area (MM/PBSA) method.

### 2.8 Experimental validation of BRSK2-associated expression patterns

Animal protocols proceeded under the ethical oversight of the Institutional Animal Care and Use Committee at Chengdu University of Traditional Chinese Medicine (Approval: 2025105). Pathogen-free male Sprague-Dawley rats (6–8 weeks, 200–220 g; Beijing Sibeifu Biotechnology Co., Ltd., Beijing, China) were maintained in a controlled vivarium environment (22 ± 2 °C, 12-hour photoperiod). For fibrotic induction, anesthetized animals received a single intratracheal administration of bleomycin (5 mg/kg; Selleck, USA, Cat. No. S1214). Sham-treated controls underwent identical vehicle instillation. Tissues were collected 28 days after induction. Three independent biological replicates per group were used for the qRT-PCR and immunofluorescence analyses reported below. Fibrosis induction was confirmed by histopathological examination. Lung tissues were fixed in 4% paraformaldehyde, embedded in paraffin, and sectioned at 4 μm thickness. Hematoxylin and eosin (H&E) staining was performed to evaluate lung architecture and inflammatory changes, while Masson’s trichrome staining was used to assess collagen deposition and fibrotic remodeling. Histological images were acquired using a light microscope and compared between bleomycin-treated and control groups.

Total RNA was extracted from lung tissues using the Animal Total RNA Isolation Kit (Foregene, China) according to the manufacturer’s instructions. Complementary DNA (cDNA) was synthesized using a reverse transcription kit (Servicebio, China), and quantitative real-time PCR (qRT-PCR) was performed using SYBR Green chemistry (Servicebio, China) on a Bio-Rad CFX Connect real-time PCR detection system. Expression levels of MUC5B, TOLLIP, BRSK2, FOXA2, CDC25B, PKD1, WEE1, and MYC were quantified. Relative expression levels were normalized to β-Actin and calculated using the 2^-^ΔΔCt method. Primer sequences are provided in **Supplementary Table 2**.

To evaluate protein-level changes in selected BRSK2-associated network features, immunofluorescence staining was performed on paraffin-embedded lung tissue sections. Following deparaffinization, antigen retrieval, and blocking, sections were incubated overnight at 4 °C with primary antibodies against BRSK2 (Proteintech, Cat. No. 00180086), FOXA2 (Abmart, Cat. No. 354382), and CDC25B (Affbiotech, Cat. No. 605328C). After washing, sections were incubated with species-specific fluorescent secondary antibodies and counterstained with DAPI. Fluorescence images were acquired using a confocal fluorescence microscope. Protein expression patterns and localization of BRSK2, FOXA2, and CDC25B were compared between fibrotic and control lung tissues as expression-level validation of the computationally prioritized features.

### 2.9 Statistical analysis

All statistical analyses were performed using R (v4.3.1). Unless otherwise specified, two-group comparisons were conducted using the Wilcoxon rank-sum test or Student’s t-test as appropriate. Correlations were evaluated using Pearson correlation analysis. For TF–target network screening, nominal P < 0.05 together with |r| > 0.4 was used as the predefined selection criterion; Benjamini–Hochberg correction was applied to high-dimensional differential and enrichment analyses as specified in the corresponding sections. Statistical significance was defined as a two-sided P < 0.05.

## 3 Results

### 3.1 Multi-omics prioritize candidate genes associated with IPF susceptibility

SMR analyses integrating IPF GWAS summary statistics with mQTL, eQTL, and pQTL datasets identified multiple molecular traits at the chromosome 11p15.5 susceptibility locus that were associated with IPF risk (**Table 1**). Nine CpG sites showed significant SMR associations after multiple-testing correction, suggesting genetically supported relationships between DNA methylation and IPF susceptibility at this locus. Integration with eQTL data further prioritized MUC5B, TOLLIP, and BRSK2. Whereas MUC5B and TOLLIP represent established IPF susceptibility genes, BRSK2 emerged as a comparatively underexplored candidate supported by significant expression-level SMR evidence.

**Table 1:** Significant SMR associations between molecular traits at chromosome 11p15.5 and IPF susceptibility.

QTL	Probe	B_SMR	P_SMR	P_HEIDI	FDR
mQTL	cg07497941	-1.03	5.47E-06	0.65	4.30E-04
	cg09109553	0.76	2.62E-05	0.84	0.02
	cg16793525	0.52	3.50E-07	0.01^‡^	4.30E-04
	cg25931491	0.60	1.76E-05	0.11	0.01
	cg14306344	0.57	6.68E-07	0.02	7.43E-04
	cg23372535	0.57	9.26E-07	0.09	1.02E-03
	cg07652408	0.70	9.40E-06	0.55	7.61E-03
	cg09167291	0.53	4.10E-07	0.05	4.78E-04
	cg16022876	0.41	1.01E-07	0.02	1.50E-04
eQTL	MUC5B	1.46	4.29E-09	–	1.48E-05
	BRSK2	0.47	8.29E-06	0.04	5.19E-03
	TOLLIP	-1.63	5.14E-11	0.02	8.02E-07
pQTL	BRSK2	0.45	1.11E-10	1.76e-06^†^	2.46E-07

^†^This association did not pass the HEIDI test and was not considered convergent protein-level genetic evidence. ‡The displayed P_HEIDI value was rounded to two decimal places; the unrounded value was >0.01 and therefore passed the predefined HEIDI criterion. QTL, quantitative trait locus; mQTL, methylation quantitative trait locus; eQTL, expression quantitative trait locus; pQTL, protein quantitative trait locus; SMR, summary-data-based Mendelian randomization; HEIDI, heterogeneity in dependent instruments; FDR, false discovery rate; B_SMR, beta coefficient from SMR analysis; P_SMR, P-value for SMR test; P_HEIDI, P-value for HEIDI test.

Most molecular traits with available HEIDI results showed *P_HEIDI_*>0.01, consistent with associations that may arise from a shared genetic signal rather than linkage between distinct causal variants. However, the BRSK2 pQTL association did not pass the HEIDI test (*P_HEIDI_* = 1.76e-06) and was therefore not interpreted as independent protein-level evidence for a shared causal variant. The HEIDI result was unavailable for the MUC5B eQTL association, precluding assessment of heterogeneity for that signal. Cross-molecular-layer analyses integrating mQTL and eQTL data identified additional relationships between methylation and gene-expression traits within the locus (**Supplementary Table 3**). These findings support shared genetic regulation across epigenetic and transcriptional layers but do not, by themselves, establish a directional effect of DNA methylation on gene expression. Collectively, the analyses recovered the established MUC5B and TOLLIP signals and prioritized BRSK2 as a candidate gene at 11p15.5 for subsequent transcriptomic, cellular, spatial, and experimental evaluation.

### 3.2 Independent validation of candidate gene expression and DNA methylation

**Figure 1:**
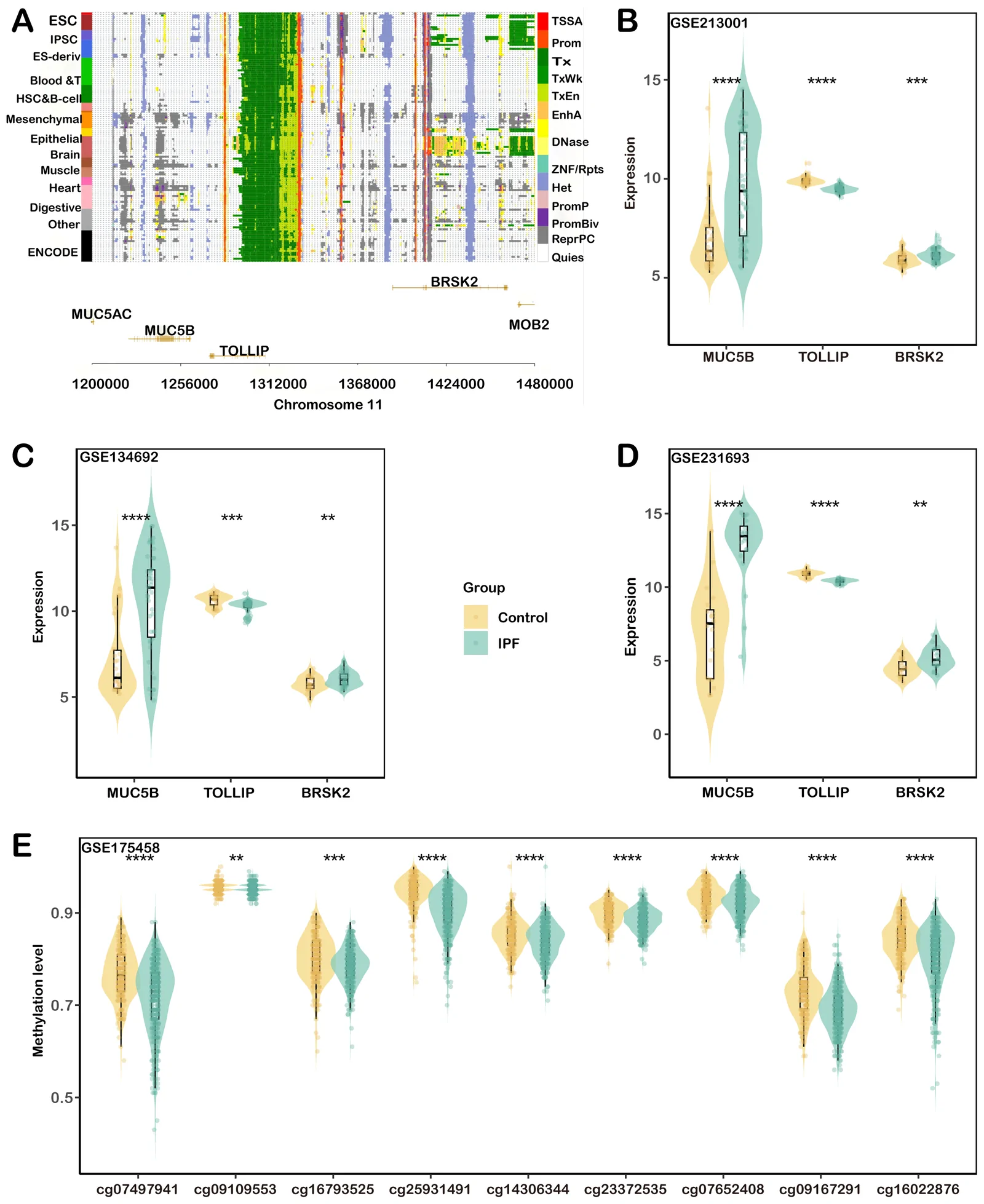
Multi-layered regulatory features of *MUC5B*, *TOLLIP*, and *BRSK2* in IPF. **(A)** Chromatin state annotations from ENCODE and Roadmap Epigenomics (ChromHMM 15-state). **(B–D)** Expression levels of *MUC5B*, *TOLLIP*, and *BRSK2* in IPF and control lung tissues across independent cohorts GSE213001 **(B)**, GSE134692 **(C)**, and GSE231693 **(D)**. **(E)** DNA methylation levels at associated CpG probes (GSE175458). *P < 0.05; **P < 0.01; ***P < 0.001; ****P < 0.0001.

MUC5B, TOLLIP, and BRSK2 were located within the chromosome 11p15.5 susceptibility locus and occupied closely clustered genomic positions (**Figure 1A**). Functional genomic annotation identified multiple promoter- and enhancer-associated chromatin states across this region, indicating a complex regulatory landscape surrounding these genes. Their molecular alterations were subsequently evaluated in independent transcriptomic and DNA-methylation datasets derived from IPF and control lung tissues. Consistent with the directions of the corresponding eQTL-based SMR estimates, MUC5B and BRSK2 expression was significantly increased in IPF lung tissues (*P* < 0.0001 and *P* < 0.001, respectively), whereas TOLLIP expression was significantly decreased (*P* < 0.0001) (**Figures 1B–D**).

Differential methylation analysis identified significant alterations at seven CpG sites within the BRSK2 locus—cg07652408, cg14306344, cg16022876, cg23372535, cg16793525, cg25931491, and cg09167291(all P < 0.0001) —as well as at cg07497941 near MUC5B (*P* < 0.0001) and cg09109553 associated with TOLLIP (*P* < 0.01) (**Figure 1E**). These findings demonstrate that the prioritized genes are accompanied by reproducible transcriptional and epigenetic alterations in independent IPF cohorts. Together with the SMR results, the data support MUC5B, TOLLIP, and BRSK2 as candidate genes associated with the 11p15.5 susceptibility locus, while the functional relationships between individual CpG sites and gene expression require further investigation.

### 3.3 Integrated regulatory networks of candidate IPF genes

**Figure 2:**
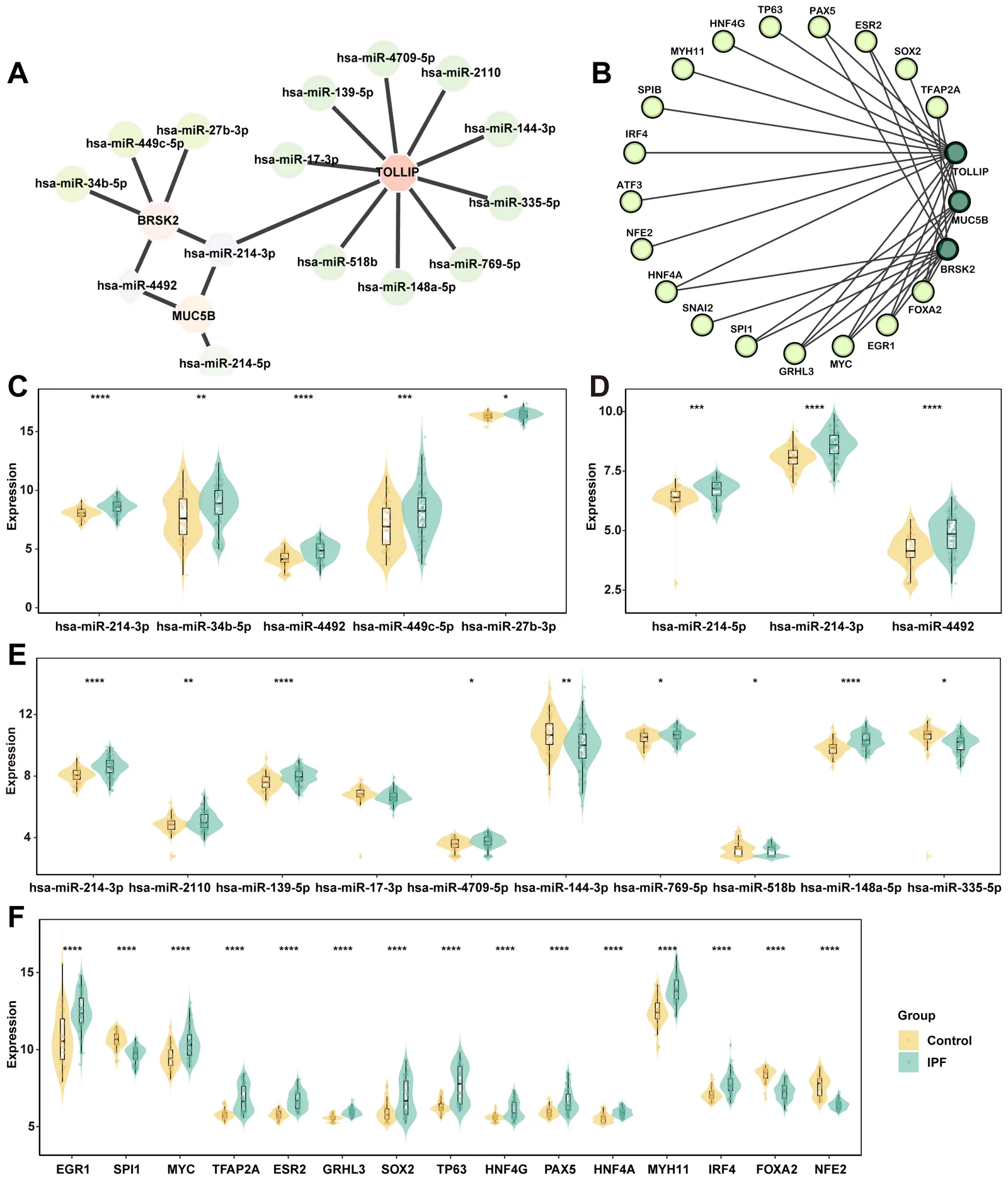
Integrated miRNA and transcription factor networks associated with candidate IPF genes. **(A)** MicroRNA regulatory network centered on *MUC5B*, *TOLLIP*, and *BRSK2*. **(B)** Transcription factor (TF) regulatory network. **(C–E)** Expression levels of targeting microRNAs associated with *BRSK2* **(C)**, *TOLLIP* **(D)**, and *MUC5B* **(E)** in IPF versus healthy controls. **(F)** Differential expression of the hub TFs in IPF compared to controls. *P < 0.05; **P < 0.01; ***P < 0.001; ****P < 0.0001.

To characterize the putative regulatory architecture of the 11p15.5 candidate genes, we integrated predicted microRNA (miRNA)–target relationships, transcription factor (TF) information, and gene-expression evidence. Differential miRNA profiling revealed distinct expression patterns between IPF and control lungs (**Figure 2A**). Predicted miRNA–target analysis indicated that MUC5B and BRSK2 shared several candidate miRNAs, including hsa-miR-214-3p, hsa-miR-214-5p, hsa-miR-4492, and hsa-miR-449c-5p, all of which were significantly increased in IPF samples (**Figures 2C, D**; all *P* < 0.001). TOLLIP was associated with a different set of candidate miRNAs, including increased hsa-miR-139-5p and hsa-miR-148a-5p and decreased hsa-miR-144-3p and hsa-miR-335-5p (**Figure 2E**; all *P* < 0.05).

TF-based network analysis identified multiple candidate transcriptional regulators associated with the three genes (**Figure 2B**). EGR1, MYC, and TFAP2A were significantly increased in IPF, whereas SPI1, FOXA2, and NFE2 were decreased (**Figure 2F**; **Supplementary Figures 1G, H**; *P* < 0.0001). Pearson correlation analysis in GSE213001 further revealed gene-specific association patterns. MUC5B expression was positively correlated with TFAP2A and SOX2; TOLLIP expression was positively correlated with FOXA2 and NFE2 but negatively correlated with EGR1 and MYC; and BRSK2 expression was positively correlated with EGR1, MYC, and TFAP2A and negatively correlated with SPI1 (**Supplementary Figure 2**). Together, these results identify dataset-supported miRNA- and TF-associated regulatory relationships surrounding the 11p15.5 candidate genes, although direct regulatory effects require experimental validation.

**Figure 3:**
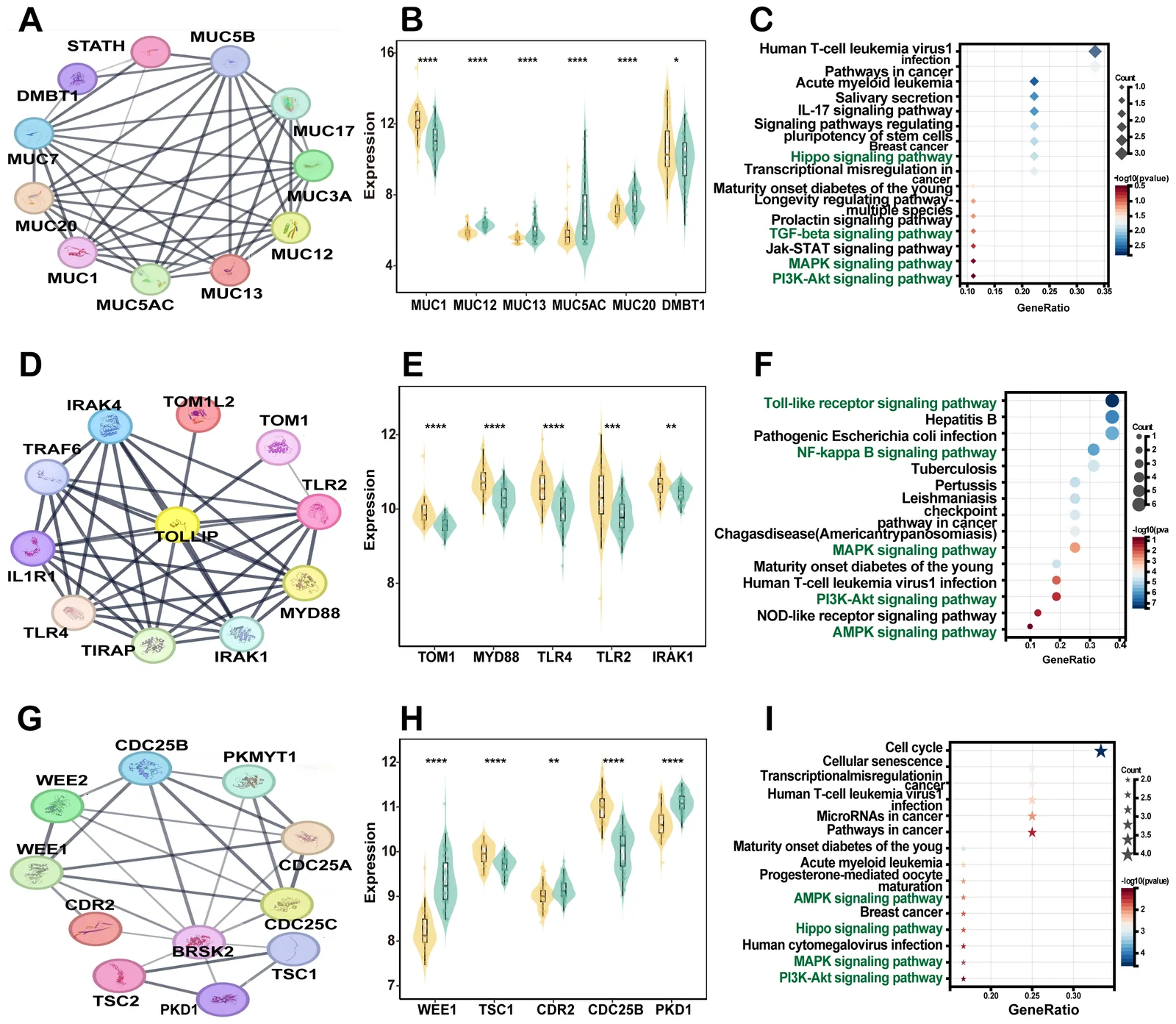
PPI networks and pathway enrichment associated with candidate genes. **(A, D, G)** PPI networks of *MUC5B* **(A)**, *TOLLIP* **(D)**, and *BRSK2* **(G)**. **(B, E, H)** Expression of interacting partners. **(C, F, I)** KEGG pathway enrichment. *P < 0.05; **P < 0.01; ***P < 0.001; ****P < 0.0001.

To further characterize the molecular contexts of the candidate genes, STRING-based protein–protein interaction networks were constructed and their network members were evaluated in independent IPF transcriptomic datasets. The MUC5B-centered network contained a mucin-associated module, in which MUC12, MUC13, and MUC5AC were significantly increased in IPF lung tissues (**Figures 3A, B**; **Supplementary Figures 1C, D**; all *P* < 0.0001). The TOLLIP-centered network included multiple components of innate immune signaling. Among these, IL1R1 expression was increased, whereas TOM1, MYD88, TLR2, TLR4, and IRAK1 were decreased in IPF samples (**Figures 3D, E**; **Supplementary Figures 1E, F**; all *P* < 0.01). The BRSK2-centered network contained proteins associated with cell-cycle control, metabolic regulation, and cellular stress responses. WEE1 and PKD1 were increased, whereas TSC1 and CDC25B were decreased in IPF lung tissues (**Figures 3G, H**; **Supplementary Figures 1A, B**; all *P* < 0.01).

KEGG enrichment analysis further revealed distinct functional contexts for the three gene-centered networks. The MUC5B-associated network was enriched in developmental and epithelial-associated signaling pathways, including Hippo signaling and signaling pathways regulating pluripotency of stem cells (**Figure 3C**). The TOLLIP-associated network was enriched in innate immune and inflammatory pathways, including Toll-like receptor, NF-κB, and NOD-like receptor signaling (**Figure 3F**). In contrast, the BRSK2-associated network was enriched in pathways related to cell-cycle regulation, cellular senescence, and AMPK signaling (**Figure 3I**). Collectively, these findings place MUC5B, TOLLIP, and BRSK2 within distinct IPF-associated molecular contexts involving epithelial remodeling, innate immune signaling, and cell-cycle and metabolic-stress regulation, respectively. The BRSK2-centered network was subsequently subjected to machine-learning analysis to prioritize candidate components for downstream validation.

### 3.4 Machine learning identifies key regulatory features associated with IPF

**Figure 4:**
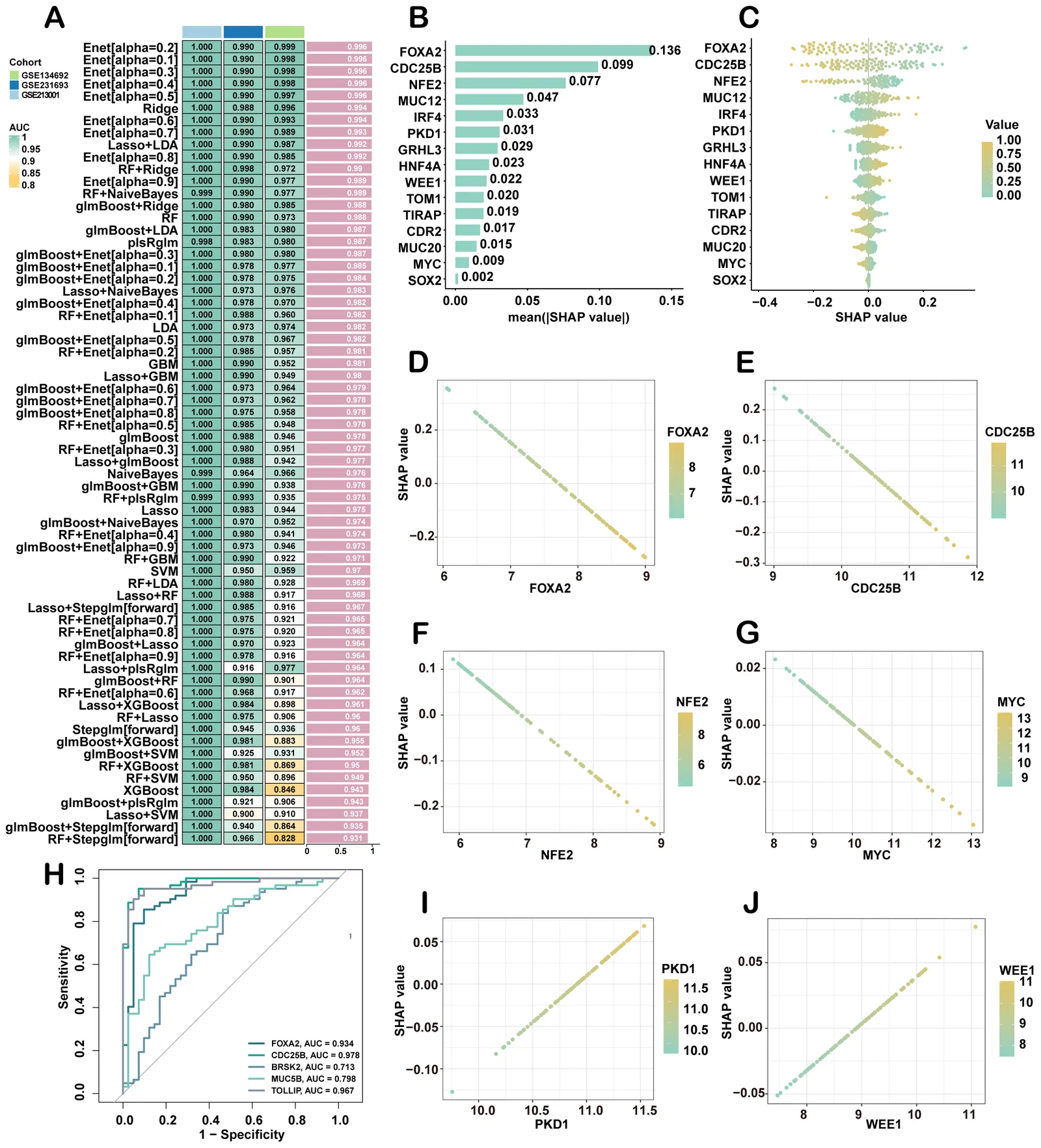
Machine learning-based feature analysis in IPF. **(A)** Performance (AUC) of machine learning algorithms across three cohorts. **(B, C)** SHAP feature importance **(B)** and summary plot **(C)**. **(D–G, I, J)** SHAP dependence plots for *FOXA2* **(D)**, *CDC25B* **(E)**, *NFE2* **(F)**, *MYC* **(G)**, *PKD1* **(I)**, and *WEE1* **(J)**. **(H)** ROC curves of selected key features.

To prioritize regulatory features within the integrated MUC5B-, TOLLIP-, and BRSK2-associated networks, we applied a multi-algorithm machine-learning framework across GSE134692, GSE231693, and GSE213001. Several model configurations, including Elastic Net, Ridge, and Lasso combined with linear discriminant analysis, showed strong cross-cohort discrimination, with AUC values exceeding 0.90 in each of the three transcriptomic cohorts (**Figure 4A**).

SHapley Additive exPlanations (SHAP) analysis was used to interpret the selected model. FOXA2, CDC25B, and NFE2 were the three most influential features, with mean absolute SHAP values of 0.136, 0.099, and 0.077, respectively (**Figure 4B**). SHAP dependence analyses indicated that lower expression of these genes shifted model predictions toward the IPF class (**Figures 4C–F**). In contrast, higher expression of MUC12 and PKD1 contributed positively to IPF prediction, whereas MYC and WEE1 showed additional feature-specific SHAP patterns (**Figures 4B, C, G, I, J**).

Exploratory single-gene ROC analyses showed high discriminatory capacity for CDC25B (AUC = 0.978), FOXA2 (AUC = 0.934), and TOLLIP (AUC = 0.967), whereas MUC5B and BRSK2 showed more moderate performance (AUC = 0.798 and 0.713, respectively) (**Figure 4H**). Collectively, these analyses prioritized FOXA2, CDC25B, and NFE2 as key features within the integrated regulatory network and indicated that multigene regulatory patterns provided stronger separation of IPF and control samples than individual candidate genes alone.

### 3.5 Cellular and spatial context of the regulatory network in IPF

**Figure 5:**
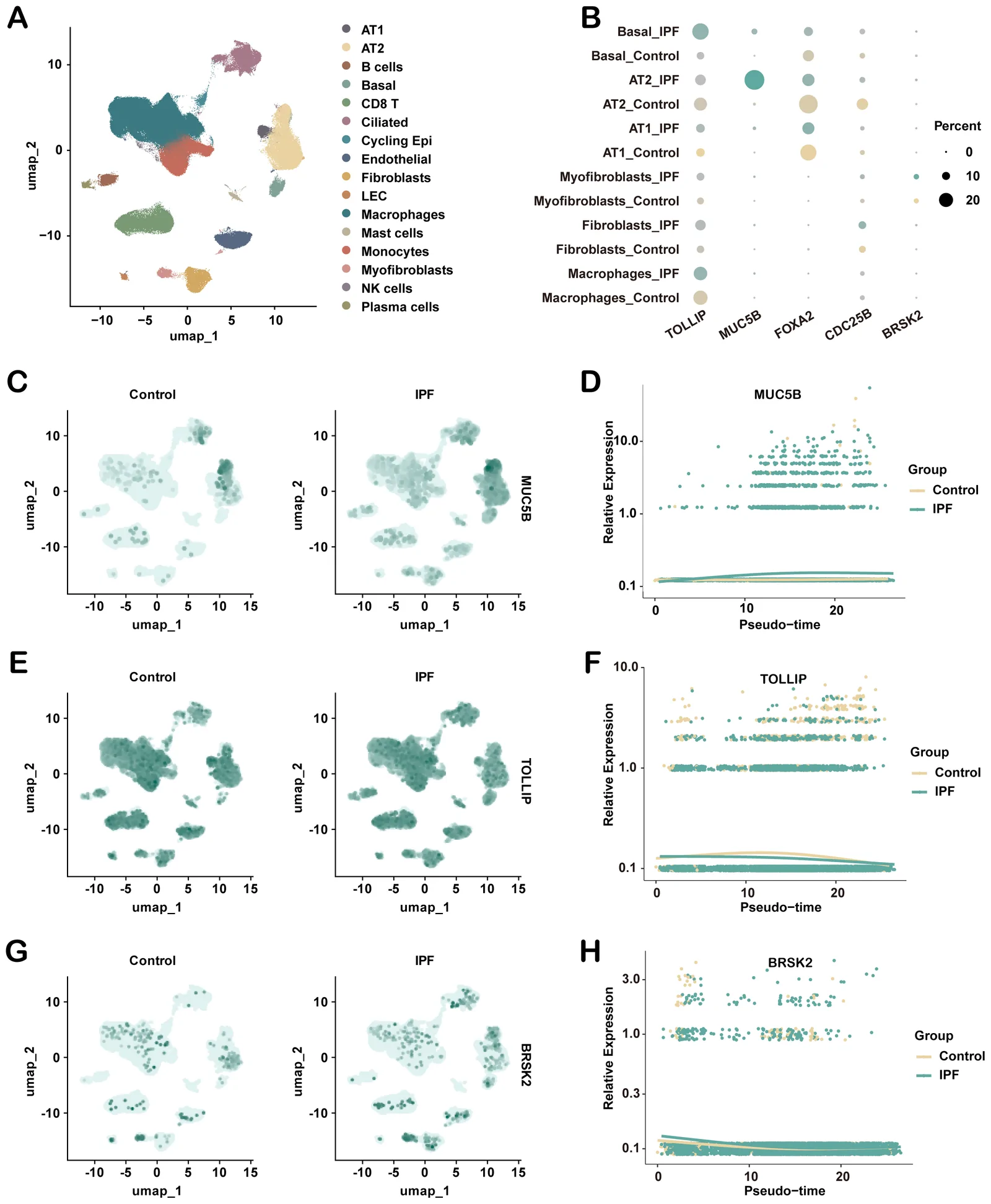
Single-cell expression profiles and pseudotime-associated patterns of candidate genes. **(A)** UMAP visualization of identified cell clusters. **(B)** Dot plot showing the expression of selected key genes across cell populations in IPF and controls. **(C, E, G)** UMAP plots displaying the expression distribution of *MUC5B* **(C)**, *TOLLIP* **(E)**, and *BRSK2* **(G)** in control and IPF lungs. **(D, F, H)** Relative expression of *MUC5B* **(D)**, *TOLLIP* **(F)**, and *BRSK2* **(H)** along the inferred pseudotime trajectory.

Integrated scRNA-seq analysis identified the major epithelial, stromal, and immune cell populations in control and IPF lungs based on canonical marker expression (**Figure 5A**; **Supplementary Figure 3E**). Quality-control metrics, UMAP visualization by disease condition and study origin, and study-specific cell-type contributions were examined to characterize the composition and integration of the combined dataset (**Supplementary Figures 3A–D**). MUC5B was predominantly expressed in alveolar epithelial populations, particularly AT2 cells, whereas TOLLIP showed a broader distribution across macrophage, epithelial, and stromal populations (**Figures 5B, C, E**). In contrast, BRSK2 was preferentially expressed in fibroblasts and myofibroblasts (**Figures 5B, G**; **Supplementary Figure 3F**). The expression patterns of DCN, COL1A1, ACTA2, TAGLN, and POSTN further supported the mesenchymal and profibrotic identity of these populations (**Supplementary Figures 3F–K**). The machine-learning-prioritized genes CDC25B and FOXA2 also exhibited distinct cell-type-associated expression patterns (**Figure 5B**).

Pseudotime analysis further revealed gene-specific expression patterns across computationally ordered cellular states. MUC5B showed an increasing expression trend along the inferred pseudotime ordering, whereas TOLLIP generally showed a decreasing trend. (**Figures 5D, F**) BRSK2 also showed a modest increasing trend across pseudotime (**Figure 5H**). These patterns were interpreted descriptively as associations with cellular-state organization rather than as formal IPF-versus-control comparisons or evidence of longitudinal disease progression.

**Figure 6:**
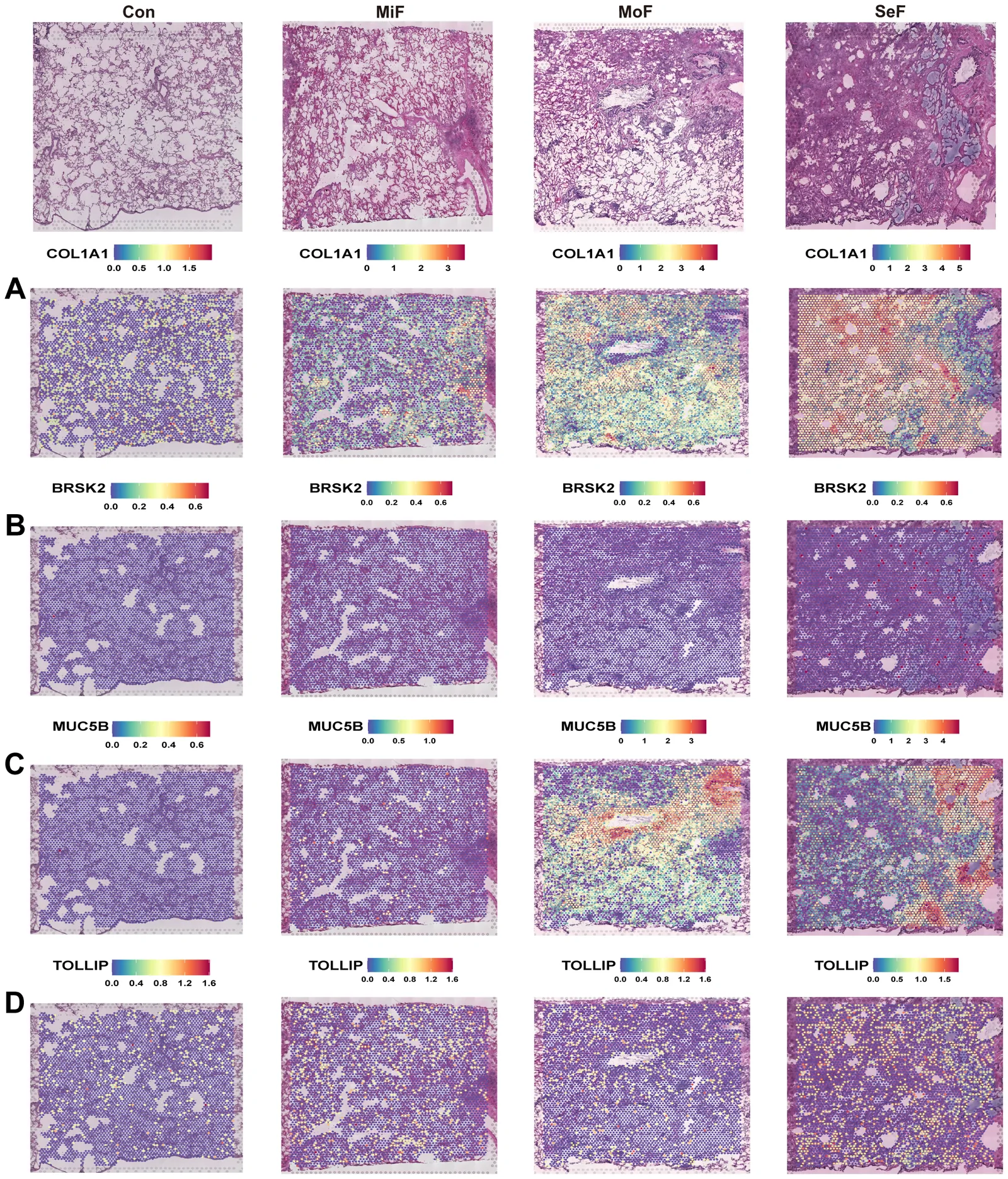
Spatial mapping of candidate genes across regional fibrosis-severity categories. **(A–D)** Spatial transcriptomic visualizations displaying the expression and distribution of *COL1A1* **(A)**, *BRSK2* **(B)**, *MUC5B* **(C)**, and *TOLLIP* **(D)** in representative lung sections across control (Con), mild (MiF), moderate (MoF), and severe fibrosis (SeF) regional fibrosis categories.

Spatial transcriptomic analysis was then used to examine these genes across histologically defined control (Con) and mild (MiF), moderate (MoF), and severe (SeF) regional fibrosis categories. MUC5B showed limited spatial autocorrelation in control tissue but stronger regional clustering with increasing regional fibrosis severity across MiF, MoF, and SeF sections, with Moran’s *I* increasing from approximately 0.03 to 0.77 (*P* < 0.0001, **Figures 6A, C**; **Supplementary Table 4**). BRSK2 expression was relatively limited in control and mildly fibrotic regions but was more prominent in moderate and severely fibrotic regions (**Figure 6B**), consistent with its preferential expression in fibroblast and myofibroblast populations. TOLLIP displayed a broader and distinct spatial pattern across the regional fibrosis categories (**Figure 6D**). Together, the single-cell and spatial analyses placed MUC5B, TOLLIP, and BRSK2 within epithelial, immune-associated, and mesenchymal contexts, respectively. Their severity-associated spatial distributions further support distinct molecular contexts across regionally heterogeneous IPF lung tissue.

### 3.6 Computational assessment of the druggability of the BRSK2-associated network

To explore the pharmacological tractability of BRSK2 and its associated network, candidate compounds were collected through database mining and literature curation. Of the 25 compounds identified, 19 showed favorable predicted pharmacokinetic properties according to SwissADME analysis (**Supplementary Table 5**). Integration of SwissTargetPrediction results with the BRSK2-associated network identified four overlapping predicted targets: CDC25B, HNF4A, WEE1, and ESR2. Among these, CDC25B and WEE1 were prioritized for subsequent structural analyses based on their network associations and relevance to the BRSK2-centered regulatory module (**Supplementary Figure 4A**).

Molecular docking identified several compounds with favorable predicted binding scores. GW296115 and staurosporine showed the highest docking scores for BRSK2 (both −9.1 kcal/mol), whereas camptothecin, luteolin, apigenin, genistein, kaempferol, chrysin, fisetin, and quercetin showed scores ranging from −8.0 to −8.3 kcal/mol. Camptothecin also showed predicted binding to CDC25B, while curcumin and resveratrol showed predicted interactions with WEE1. The modeled complexes involved hydrogen-bond and hydrophobic contacts, including interactions with Lys49 and Lys28 in the BRSK2 kinase domain (**Supplementary Figures 4**-**6**).

**Figure 7:**
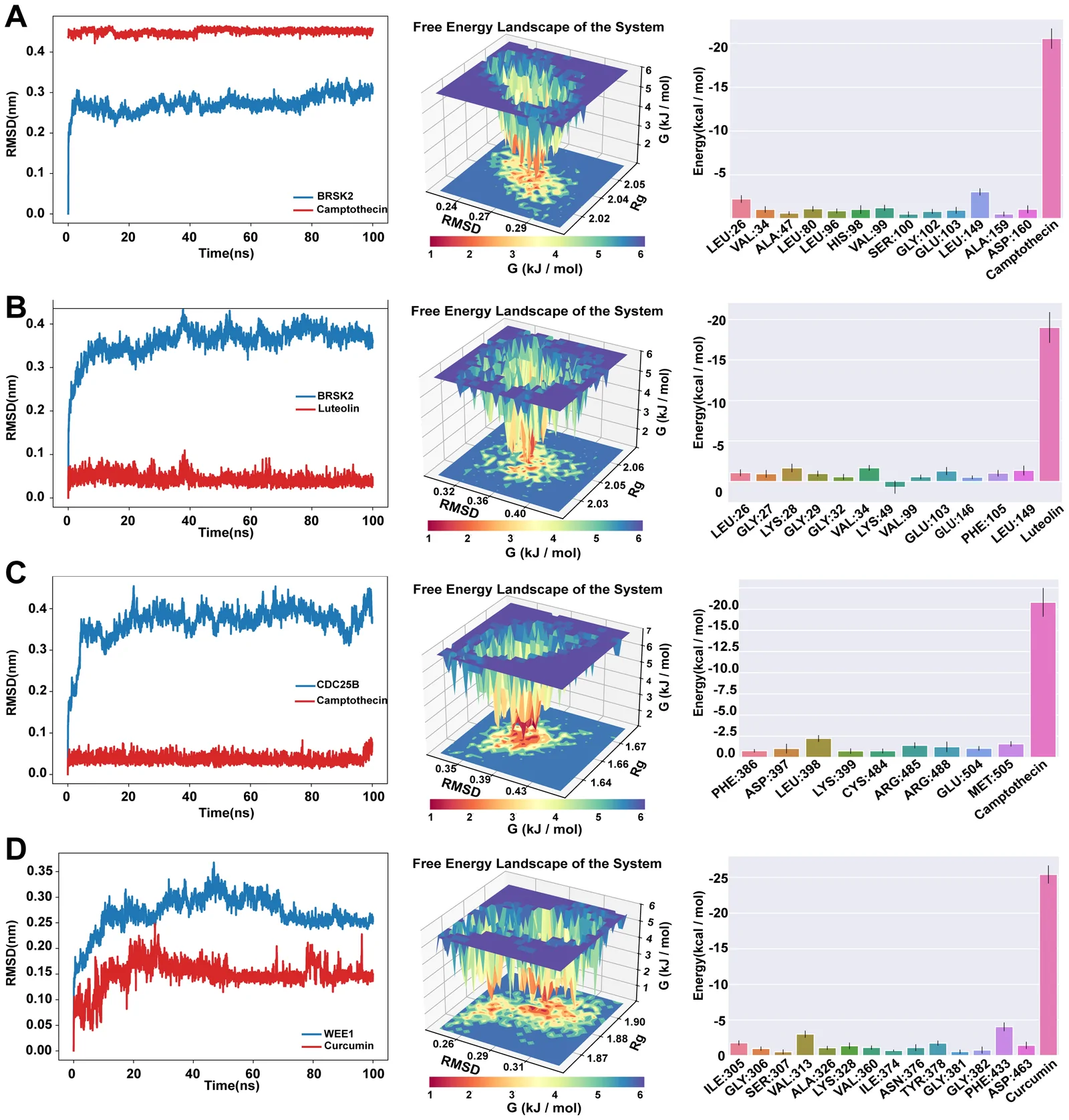
Molecular dynamics (MD) simulations and stability validation of the core protein-ligand complexes. **(A–D)** Root-mean-square deviation (RMSD) trajectories over 100 ns (left), 3D Free Energy Landscape (FEL) plots (middle), and per-residue free energy decomposition bar charts (right) for the BRSK2–camptothecin **(A)**, BRSK2–luteolin **(B)**, CDC25B–camptothecin **(C)**, and WEE1–curcumin **(D)** complexes, illustrating stable conformational dynamics and key energetic contributors.

Representative protein–ligand complexes were further evaluated using 100-ns molecular dynamics simulations. The RMSD profiles of the BRSK2–camptothecin and BRSK2–luteolin complexes stabilized after approximately 20 ns and remained relatively consistent during the remaining simulation period. Free-energy landscape and residue-level energy decomposition analyses further implicated Lys49 and Lys28 in the predicted ligand-binding interfaces (**Figures 7A, B**). Comparable computational stability was observed for the CDC25B–camptothecin and WEE1–curcumin complexes (**Figures 7C, D**). Together, these analyses identify candidate small-molecule interactions with BRSK2 and selected network-associated proteins. These findings provide hypothesis-generating computational support for the pharmacological tractability of the BRSK2-associated network but require biochemical binding assays, target-engagement studies, and functional validation in fibrotic models.

### 3.7 Experimental validation of BRSK2-associated expression patterns in pulmonary fibrosis

**Figure 8:**
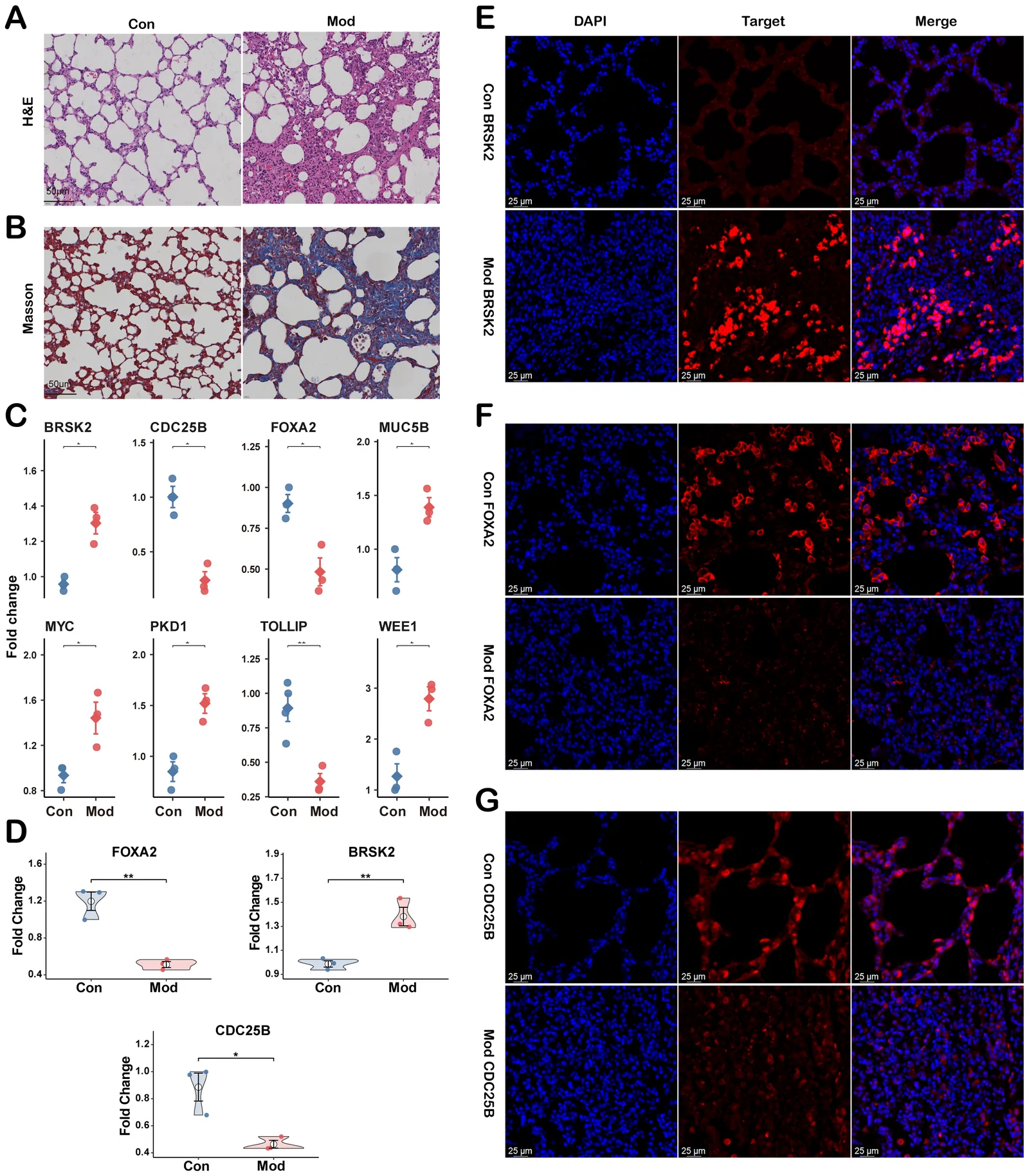
Validation of BRSK2-centered regulatory targets in bleomycin-induced pulmonary fibrosis. **(A, B)** Representative images of H&E **(A)** and Masson’s trichrome **(B)** staining of lung tissues from the Con and Mod groups. Scale bars = 50 μm (black bars). **(C)** RT-qPCR quantification of relative mRNA expression for BRSK2, CDC25B, FOXA2, MUC5B, MYC, PKD1, TOLLIP, and WEE1 (n = 3 per group). Data calculated using the 2^-^ΔΔCt method. **(D)** Statistical quantification of relative protein levels for FOXA2, BRSK2, and CDC25B based on mean immunofluorescence intensity (n=3). **(E–G)** Representative immunofluorescence images showing expression and localization of BRSK2 **(E)**, FOXA2 **(F)**, and CDC25B **(G)**. Nuclei stained with DAPI (blue); target proteins labeled in red. Scale bars = 25 μm (white bars). Data are presented as mean ± SEM. *p < 0.05, **p < 0.01 versus the Con group.

Histopathological changes in bleomycin-induced pulmonary fibrosis were evaluated using hematoxylin and eosin and Masson’s trichrome staining. Compared with the control (Con) group, the model (Mod) group exhibited alveolar wall thickening, inflammatory cell infiltration, and increased interstitial collagen deposition, consistent with successful induction of pulmonary fibrosis (**Figures 8A, B**).

We next examined the expression of BRSK2 and selected network-associated genes in fibrotic lung tissues. RT–qPCR analysis showed that BRSK2, MUC5B, MYC, PKD1, and WEE1 were significantly increased in the Mod group, whereas CDC25B, FOXA2, and TOLLIP were decreased relative to controls (**Figure 8C**). These expression directions were consistent with those observed in the preceding transcriptomic and network analyses.

Immunofluorescence staining was subsequently performed to evaluate BRSK2, FOXA2, and CDC25B at the protein level. Quantitative analysis showed increased BRSK2 abundance in fibrotic lung tissues, accompanied by reduced FOXA2 and CDC25B signals (**Figures 8D–G**). Together, these experiments confirmed fibrosis-associated alterations in BRSK2 and selected network-associated features at the transcript and protein levels. However, the observed expression patterns do not establish direct regulatory relationships among these molecules. These findings provide experimental support for the association of BRSK2 with pulmonary fibrotic remodeling and support its prioritization for subsequent functional investigation.

## 4 Discussion

In this study, we refined the molecular landscape of the chromosome 11p15.5 IPF susceptibility locus by integrating genetic, epigenetic, transcriptomic, cellular, spatial, and experimental evidence. In addition to recovering the established MUC5B and TOLLIP signals, our analyses prioritized BRSK2 as a comparatively underexplored candidate associated with fibroblast- and myofibroblast-enriched remodeling programs. The regulatory-network and machine-learning analyses further placed BRSK2 within a molecular context involving cell-cycle control, metabolic stress, and proliferative signaling. Importantly, these findings support the biological prioritization and contextualization of BRSK2 but do not establish it as a causal effector gene or direct driver of pulmonary fibrosis.

The close genomic proximity of MUC5B, TOLLIP, and BRSK2 within the telomeric chromosome 11p15.5 region highlights the complexity of assigning non-coding IPF susceptibility signals to individual effector genes. Previous studies have identified partially independent association signals involving MUC5B and TOLLIP, whereas conditional genetic analyses have indicated a more complex architecture across this locus (25, 26). In the present study, the eQTL-based SMR association for BRSK2 passed the HEIDI test and was accompanied by consistent expression and methylation alterations in independent IPF cohorts. However, the corresponding BRSK2 pQTL association showed significant HEIDI heterogeneity and was therefore not interpreted as independent protein-level evidence for a shared causal variant. More broadly, the physical proximity of these genes should not be considered evidence that they are regulated by the same causal variant or chromatin domain. Non-coding variants may exert context-dependent effects on multiple genes, and the available SMR and transcriptomic data cannot resolve the three-dimensional regulatory architecture of this region. Fine-mapping, allele-specific regulatory analyses, and chromatin-conformation studies will therefore be required to determine whether MUC5B, TOLLIP, and BRSK2 are functionally connected through shared or partially independent cis-regulatory mechanisms.

The three candidate genes were embedded within distinct molecular contexts. The MUC5B-centered network contained several mucin-related proteins and was enriched in epithelial and developmental pathways, consistent with its established association with epithelial dysfunction in IPF. The TOLLIP-centered network included multiple components of Toll-like receptor, NF-κB, and NOD-like receptor signaling, supporting its relationship with innate immune regulation. In contrast, the BRSK2-centered network was associated with cell-cycle regulation, cellular senescence, AMPK signaling, and stress-response pathways and included proteins such as CDC25B, WEE1, PKD1, and TSC1. The miRNA and transcription-factor analyses further identified candidate regulatory associations surrounding these networks. However, these relationships were derived from database predictions, protein-interaction resources, and expression correlations and should not be interpreted as experimentally confirmed regulatory interactions. In particular, the observed correlations between BRSK2 and transcriptional features such as EGR1, MYC, TFAP2A, and SPI1 do not establish direct transcriptional regulation.

Machine learning was used primarily as a feature-prioritization strategy rather than as a clinical diagnostic framework. Across complementary algorithms, FOXA2, CDC25B, and NFE2 consistently contributed to the discrimination between IPF and control transcriptomic profiles, and FOXA2 and CDC25B were subsequently evaluated experimentally. The lower expression of these genes shifted model predictions toward the IPF class, whereas other network components, including MUC12 and PKD1, showed positive contributions to IPF prediction. These results support the prioritization of FOXA2, CDC25B, and NFE2 within the integrated network but do not establish that they are directly regulated by BRSK2 or that they have immediate clinical biomarker utility. Their high single-gene AUC values should also be interpreted cautiously because retrospective transcriptomic classification may be influenced by cohort composition, disease severity, tissue sampling, and platform-specific effects. Validation in larger donor-level cohorts and mechanistic models will be necessary to determine their biological and translational relevance.

Mechanistically, BRSK2 belongs to the AMPK-related kinase family and has been implicated in cellular energy metabolism, stress responses, and cell-cycle regulation (27). Our analyses linked BRSK2 to CDC25B, WEE1, MYC, and related signaling programs, placing it within a molecular network associated with checkpoint control and proliferative stress. These processes are relevant to fibroblast activation and myofibroblast persistence in pulmonary fibrosis (28, 29). Recent work demonstrated that glutathione reductase (GSR) deficiency exacerbates oxidative stress and promotes fibrotic remodeling, further highlighting the importance of redox homeostasis and stress-response pathways in IPF pathogenesis (30).Consistent with this interpretation, an independent IPF-focused experimental study reported that BRSK2 inhibition attenuated TGF-β1-induced fibroblast activation and reduced α-SMA, collagen, and inflammatory cytokine expression (31). This evidence provides functional support for the involvement of BRSK2 in IPF-associated fibroblast remodeling, although its precise position within the TGF-β1 signaling hierarchy remains unresolved.

Beyond canonical SMAD signaling, TGF-β1-responsive fibroblast activation is also shaped by transcriptional and epigenetic regulation. Rubio et al. showed that reduced MIRLET7D and hyperactive EP300 in IPF fibroblasts disrupt HDAC1/2–MiCEE-dependent heterochromatin formation and transcriptional silencing, whereas EP300 inhibition attenuated fibrotic phenotypes (32). Complementarily, Cordero et al. demonstrated that nuclear miR-9 promotes TGF-β1-responsive transcription through H3K4me3 broadening, G-quadruplex formation, and promoter–super-enhancer looping, with nuclear miR-9 enrichment also observed in patient-derived IPF fibroblasts (33). These findings indicate that profibrotic fibroblast states involve coordinated alterations in transcriptional repression and TGF-β1-responsive gene activation. Notably, independent experimental evidence has shown that BRSK2 inhibition attenuates TGF-β1-induced fibroblast activation, accompanied by reduced α-SMA, collagen, and inflammatory cytokine expression, supporting a functional role for BRSK2 in TGF-β1-associated fibrotic remodeling. In this context, the enrichment of BRSK2 in fibroblasts and myofibroblasts observed in our study further supports its relevance to the profibrotic mesenchymal state. However, whether BRSK2 directly interfaces with the MIRLET7D/EP300-, HDAC/MiCEE-, or miR-9-associated chromatin regulatory mechanisms described above remains unresolved and warrants further mechanistic investigation.

The single-cell, spatial, and experimental analyses provided complementary biological context for the prioritized genes. MUC5B was preferentially expressed in epithelial populations (15, 34), TOLLIP showed a broader distribution across macrophage, epithelial, and stromal compartments (35), and BRSK2 was enriched in fibroblasts and myofibroblasts. Higher BRSK2 expression was also observed in IPF-associated mesenchymal states and in regions with greater histological fibrosis severity. These findings should be interpreted as cross-sectional cellular and regional associations rather than longitudinal disease progression, because pseudotime represents computational ordering and the spatial fibrosis categories were derived from distinct regions of the same IPF lungs. In bleomycin-treated lungs, increased BRSK2 expression was accompanied by reduced FOXA2 and CDC25B at the transcript and protein levels, consistent with the integrated computational findings. Nevertheless, these concordant expression changes do not establish direct regulatory relationships among the three molecules, and the bleomycin model does not fully reproduce the chronic and heterogeneous biology of human IPF.

This regional fibrosis-severity framework is conceptually related to that described by McDonough et al., who used microCT-derived alveolar surface density, histological assessment, and bulk RNA and microRNA profiling to classify spatially distinct lung samples as control, structurally preserved or minimally fibrotic IPF, moderately fibrotic IPF, and advanced fibrosis (36). However, the two classification systems are not directly interchangeable. The present study analyzed histologically selected Visium tissue blocks from the same four IPF lungs (18), whereas McDonough et al. analyzed systematically sampled tissue cores from 10 IPF and six control lungs. Both approaches leverage intrapulmonary spatial heterogeneity to characterize molecular changes associated with regional fibrosis severity, but neither represents direct longitudinal observation of the same lung region over time.

The structural analyses further suggested that BRSK2 and selected network-associated proteins may be amenable to small-molecule interactions. Docking and molecular dynamics simulations identified structurally compatible complexes involving BRSK2, CDC25B, and WEE1. However, computational binding scores and simulation stability do not demonstrate biochemical binding, target engagement, kinase inhibition, or antifibrotic efficacy. Moreover, compounds such as camptothecin, luteolin, curcumin, and staurosporine act on multiple molecular targets, and their previously reported antifibrotic effects cannot be attributed specifically to BRSK2 (37–39). These compounds should therefore be considered computationally prioritized chemical probes rather than candidate IPF therapies, pending biochemical and functional validation.

Several limitations should be acknowledged. First, SMR identifies molecular traits sharing genetic associations with disease but cannot independently establish biological causality. The BRSK2 pQTL association did not pass the HEIDI test, limiting its interpretation as protein-level genetic evidence. Second, the absence of fine-mapping and chromatin-interaction data prevents definitive assignment of the 11p15.5 signals to individual genes or regulatory elements. Third, the miRNA, TF, and PPI networks were partly based on database predictions and expression correlations and therefore require direct experimental validation. Fourth, heterogeneity across single-cell cohorts and the nested structure of the spatial samples may influence the observed cellular and regional distributions. Finally, although qPCR and immunofluorescence confirmed fibrosis-associated expression changes in our experimental model, direct BRSK2 perturbation experiments were not performed in the present study. Further studies are required to define the molecular mechanisms through which BRSK2 modulates TGF-β1-responsive fibroblast remodeling and whether it directly interfaces with the transcriptional or epigenetic mechanisms discussed above.

In conclusion, our findings refine the molecular landscape of the chromosome 11p15.5 IPF susceptibility locus and place MUC5B, TOLLIP, and BRSK2 within epithelial, immune-associated, and mesenchymal remodeling contexts, respectively. The integrated evidence prioritizes BRSK2 as a candidate component of an IPF-associated profibrotic fibroblast state. Its causal contribution, relationship with TGF-β1-responsive chromatin regulation, and pharmacological tractability nevertheless require targeted mechanistic validation.

## Data Availability

The publicly available omics datasets analyzed in this study and their accession numbers are listed in **Supplementary Table 1**. Experimental and computational data generated in the present study are included in the article and **Supplementary Material** or are available from the corresponding author upon reasonable request.
