# Supplementary material for: A regulatory network underlying idiopathic pulmonary fibrosis
Source: Front Immunol. 2026 Aug 20;17:1919175. doi: 10.3389/fimmu.2026.1919175 (PMC13537991; doi:10.3389/fimmu.2026.1919175)
Supplement: Supplementary file 1 [file SupplementaryFile1.docx]

**A regulatory network underlying idiopathic pulmonary fibrosis**

Ganggang Li^1^, Xiaochuan Pan^1^ ^†^, Yuanyuan Wang^2^ ^†^, Peishu Lan^1^, Jie Li^1^, Xuanyu Wu^1^, Yanyun He^1^, Hongdan Wen^1^, Zhitian Cheng^1^, Quanyu Du^1*^, Wenbin Wu^1*^

1. Hospital of Chengdu University of Traditional Chinese Medicine, School of Clinical Medicine, Chengdu University of Traditional Chinese Medicine, Chengdu, 610075, China

2. Affiliated Tumor Hospital of Xinjiang Medical University, Urumqi, Xinjiang Uygur Autonomous Region 830000, China

**To whom correspondence should be addressed**: Wenbin Wu, E-mail address: [wwb1201@vip.sina.com](mailto:wwb1201@vip.sina.com).

^#^ Xiaochuan Pan and Yuanyuan Wang contributed equally to this work.

**Supplementary Figure 1.** Validation of *BRSK2*, *TOLLIP*, and *MUC5B*-associated transcription factors and interacting proteins in independent GEO datasets. (A-H) Violin plots showing the expression levels of these target-related genes between Control and IPF groups across three independent cohorts (GSE134692, GSE231693, and GSE213001). **P*<0.05*; ** P*<0.01*; *** P*<0.001*.*

**Supplementary Figure 2.** Co-expression patterns between core hub genes and predicted upstream transcription factors. (A-D) Scatter plots illustrating the correlation between the expression of *BRSK2* and its predicted transcription factors: *EGR1* (A), *MYC* (B), *TFAP2A* (C), and *SPI1* (D). (E-I) Scatter plots depicting the relationship between the expression of *MUC5B* and specific transcription factors: *ESR2* (E), *GRHL3* (F), *SOX2* (G), *TFAP2A* (H), and *SPI1* (I). (J-T) Correlation analysis of *TOLLIP* expression with specific regulatory transcription factors, including *EGR1* (J), *FOXA2* (K), *NFE2* (L), *HNF4A* (M), *HNF4G* (N), *IRF4* (O), *GRHL3* (P), *MYH11* (Q), *MYC* (R), *PAX5* (S), and *TP63* (T). Pearson correlation coefficients (R) and p-values are indicated in each panel.

**Supplementary Figure 3.** Single-cell integration, cell annotation, and mesenchymal marker expression. (A) Quality-control metrics of the integrated scRNA-seq dataset. (B, C) UMAPs colored by disease condition (B) and study origin (C). (D) Study contributions across annotated cell populations. (E) Dot plot of canonical cell-type markers. (F) Expression of BRSK2 and representative mesenchymal/fibrosis-associated markers across cell populations. (G–K) UMAP feature plots of DCN (G), COL1A1 (H), ACTA2 (I), TAGLN (J), and POSTN (K) in control and IPF lungs.

**Supplementary Figure 4.** Identification and molecular docking of BRSK2-targeted candidate compounds. (A) Venn diagram illustrating the intersection of predicted drug targets and the BRSK2 interaction network. (B) Heatmap detailing the binding energies (kcal/mol) of the top candidate compounds with BRSK2, CDC25B, and WEE1. (C-F) 3D and 2D visualizations of the optimal docking conformations and intermolecular interactions for BRSK2–camptothecin (C), CDC25B–camptothecin (D), WEE1–curcumin (E), and BRSK2–GW296115 (F).

**Supplementary Figure 5.** Molecular docking of candidate compounds with BRSK2 and related proteins. (A) Apigenin binding to BRSK2. (B) Chrysin binding to BRSK2. (C) Fisetin binding to BRSK2. (D) Genistein binding to BRSK2.

**Supplementary Figure 6.** Molecular docking of candidate compounds with BRSK2 and related proteins. (A) Kaempferol binding to BRSK2. (B) Luteolin binding to BRSK2. (C) Quercetin binding to BRSK2. (D) Resveratrol binding to WEE1.
